# *Helicobacter pylori *lipopolysaccharide modification, Lewis antigen expression, and gastric colonization are cholesterol-dependent

**DOI:** 10.1186/1471-2180-9-258

**Published:** 2009-12-14

**Authors:** Ellen Hildebrandt, David J McGee

**Affiliations:** 1 Department of Microbiology and Immunology, Louisiana State University Health Sciences Center - Shreveport, 1501 Kings Highway, Shreveport, LA 71130, USA

## Abstract

**Background:**

*Helicobacter pylori *specifically takes up cholesterol and incorporates it into the bacterial membrane, yet little is currently known about cholesterol's physiological roles. We compared phenotypes and *in vivo *colonization ability of *H. pylori *grown in a defined, serum-free growth medium, F12 with 1 mg/ml albumin containing 0 to 50 μg/ml cholesterol.

**Results:**

While doubling times were largely unaffected by cholesterol, other overt phenotypic changes were observed. *H. pylori *strain SS1 grown in defined medium with cholesterol successfully colonized the stomach of gerbils, whereas SS1 grown without cholesterol failed to colonize. *H. pylori *lipopolysaccharide often displays Lewis X and/or Y antigens. Expression of these antigens measured by whole-cell ELISA was markedly enhanced in response to growth of strain SS1, 26695, or G27 in cholesterol. In addition, electrophoretic analysis of lipopolysaccharide in wild type G27 and in mutants lacking the O-chain revealed structural changes within the oligosaccharide core/lipid A moieties. These responses in Lewis antigen levels and in lipopolysaccharide profiles to cholesterol availability were highly specific, because no changes took place when cholesterol was substituted by β-sitosterol or bile salts. Disruption of the genes encoding cholesterol α-glucosyltransferase or lipid A phosphoethanolamine transferase had no effect on Lewis expression, nor on lipopolysaccharide profiles, nor on the cholesterol responsiveness of these properties. Disruption of the lipid A 1-phosphatase gene eliminated the effect of cholesterol on lipopolysaccharide profiles but not its effect on Lewis expression.

**Conclusions:**

Together these results suggest that cholesterol depletion leads to aberrant forms of LPS that are dependent upon dephosphorylation of lipid A at the 1-position. A tentative model for the observed effects of cholesterol is discussed in which sequential steps of lipopolysaccharide biogenesis and, independently, presentation of Lewis antigen at the cell surface, depend upon membrane composition. These new findings demonstrate that cholesterol availability permits *H. pylori *to modify its cell envelope in ways that can impact colonization of host tissue *in vivo*.

## Background

*Helicobacter pylori *is a highly niche-adapted pathogen that inhabits the human stomach, is transmitted primarily within families, and has no known environmental reservoir. Chronic infections may be asymptomatic or cause gastritis, ulcer, or gastric cancer. To establish infection, the bacterium must survive transit through the acidic gastric compartment [1]. It penetrates and establishes residence in the protective mucus layer, a lipid- and cholesterol-rich environment [2,3]. Within this niche the bacterium employs a variety of mechanisms to evade host immune response.

Lipopolysaccharides (LPS) on the surface of *H. pylori *are modified to display certain human blood group antigens, primarily Lewis antigens X and Y [4-7], and less frequently H type 1, i-antigen, blood group A, or Lewis antigens A or B [8-10]. These surface LPS antigens are necessary for the establishment of infection, because mutant strains defective for LPS O-antigen synthesis or for Lewis X/Y expression fail to colonize mice [11-13]. There is evidence that Lewis antigens expressed on the bacterial surface contribute to adherence of *H. pylori *to gastric epithelial cells [10,14], and play a role in tissue tropism [15-17]. Gastric epithelial cells also express Lewis antigens [18,19], suggesting that the display of Lewis antigens on the bacterial surface may serve as a mimicry strategy. Studies of clinical isolates [18,20] and experimental infections in animals [21] support this role for bacterial Lewis antigens in immune evasion. In human infection, *H. pylori *Lewis antigens have been linked to the severity of peptic ulcer and duodenitis [16,22]. Another important feature of *H. pylori *LPS is its modified lipid A structure, with reduced acylation and fewer charged groups than is typical of enterobacteria [23]. These lipid A modifications minimize endotoxic and inflammatory properties of *H. pylori *LPS (reviewed in [24]).

Cholesterol is a nonessential nutrient for *H pylori*, though it promotes growth in serum-free media [25,26]. *H. pylori *specifically incorporate cholesterol into the bacterial membrane [27], as do a limited number of pathogenic and commensal bacteria including *Proteus mirabilis, Lactobacillus acidophilus*, *Borrelia sp*., and *Mycoplasma *[28-30]. Cholesterol may strengthen the membrane in these organisms [30-32]. *H. pylori *also uniquely form cholesterol α-glycoside [33,34], and this metabolite can be further modified by acylation or phosphatidylation [34]. Alpha-glucosylated cholesterol subverts host immune response to the bacterium in a mouse model, through suppression of phagocytosis and of T cell activation [35]. Other roles for cholesterol and cholesterol metabolites in the bacterial membrane have yet to be explored. In this report, we demonstrate that the biosynthesis of lipopolysaccharide, including Lewis antigen expression and LPS core/lipid A modification, are altered by availability of cholesterol in the growth medium. We present data indicating that these changes in the cell envelope may significantly influence the pathogen/host interaction in an animal model of infection.

## Methods

### Bacterial strains and growth conditions

Strains of *H pylori *included the laboratory strain ATCC43504 (origin: Australia), 26695 (UK), clinical isolate G27 (Italy [36], provided by N. Salama), and the mouse adapted strain SS1 (Australia; provided by Adrian Lee [37]). Bacteria were maintained at 37°C in a microaerobic atmosphere of 5% O_2_/10% CO_2 _on Campylobacter blood agar (CBA). Bacteria were passaged every 2 to 3 days, and for no more than 25 days, to minimize genetic drift. For growth in chemically defined medium [26], bacteria were inoculated from CBA into tissue culture flasks containing Ham's F12 (Gibco) with 1 mg/ml bovine serum albumin (fatty acid-free, Sigma A7906), referred to throughout as defined medium. Liquid cultures were passaged daily by dilution into fresh medium at initial densities of 1-2 × 10^6^/ml, and used at passage 3 to 5. Cell culture grade cholesterol (>99%, Sigma) was added to F12 as a stable 10× emulsion containing 500 μg/ml cholesterol dispersed in 10 mg/ml albumin, which was prepared according to [38]. The following media additions were carried out in like manner: β-sitosterol (synthetic, 95%), sodium taurocholate, sodium glycocholate, β-estradiol, progesterone (all from Sigma), dehydroepiandrosterone (Calbiochem), and β-coprostanol (Matreya).

Doubling times were determined during log phase growth by quantitating viable cells using the Cell Titer Glo reagent (Promega) as validated and described [39]. Measurement of biomass as CFU, as cellular protein, or as ATP have all produced consistent results. A value of 1 attomol ATP per cell [40] was assumed for routine passage. Possible inaccuracy of this value does not fundamentally influence interpretation of data.

**Figure 1:**
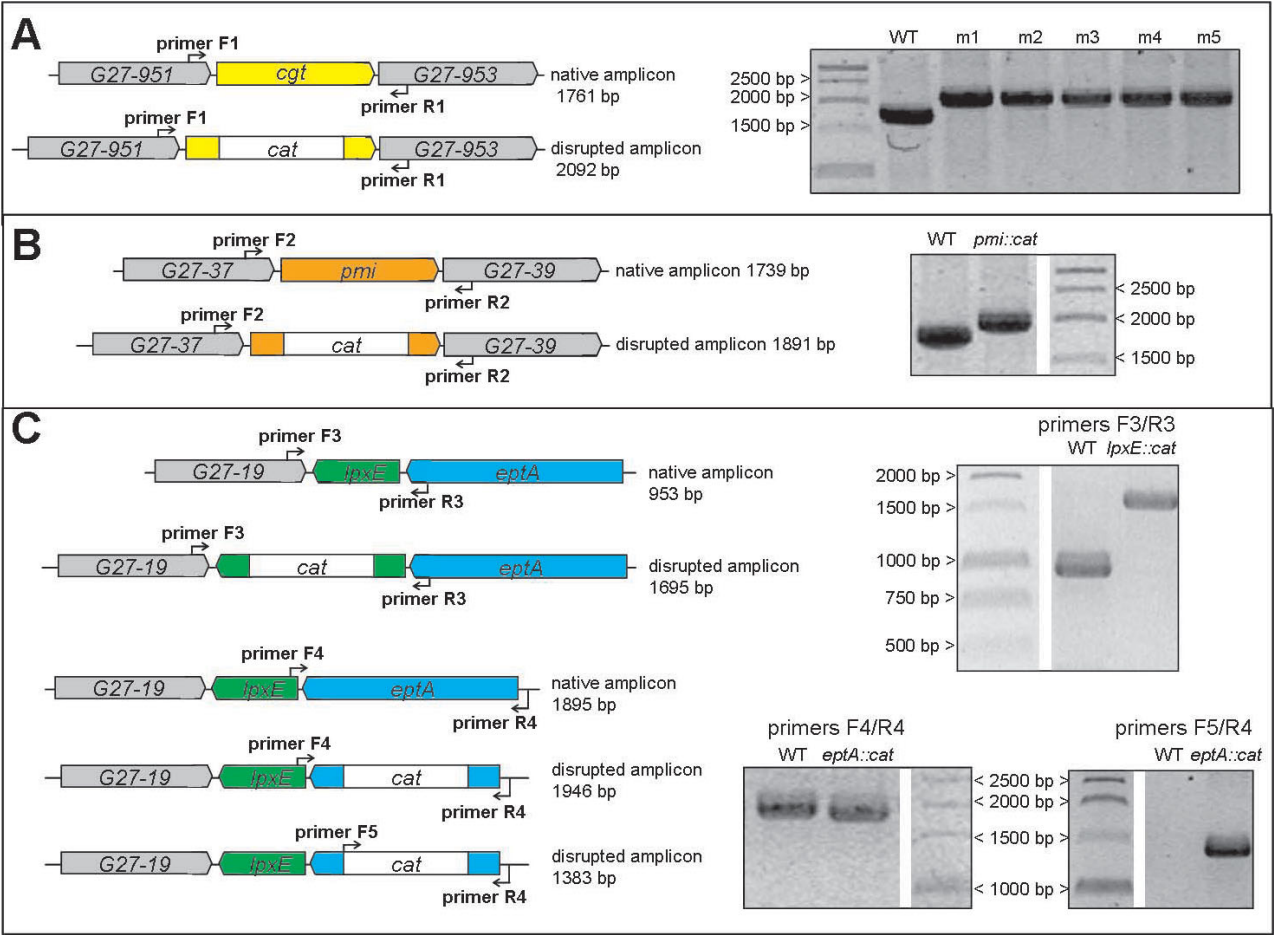
**PCR verification of allelic disruptions in *H. pylori *strain G27**. Genomic DNA was prepared from gene-disrupted G27 strains following three passages under chloramphenicol selection, then PCR amplified as shown in each scheme. Primers sequences are given in Table 1. **A. Disruption of *cgt***. Five examples are shown out of seven individual clones, all of which gave identical results in the screen. **B. Disruption of *pmi (rfbM)***. The entire chloramphenicol-resistant population was passaged in each round of selection, without clonal selection. **C. Disruption of *lpxE *and *eptA***. The entire chloramphenicol-resistant population was passaged in each round of selection, without clonal selection.

**Isogenic gene disruptions **were achieved by insertion of a *Campylobacter coli *chloramphenicol resistance element (*cat*) according to the strategy described by Chalker *et al *[41]. Primers were carefully designed so as to target sequence within open reading frames, and are listed in Table 1. Fusion PCR reactions using the PCR Extender System (5Prime) contained 2.3 nM each gel-purified template, 50 μM primer, 1× tuning buffer, 1.25 mM additional Mg^++^, 0.2 mM each dNTP, and .01 U/μl polymerase. Fusion cycle conditions were as follows: 94°C 2.5 min, 10 cycles [94°C 15 sec, 45°C 60 sec, 68°C 60 sec per kb], 25 cycles [94°C 15 sec, primer-specific Tm 30 sec, 68°C 60 sec per kb], final extension 68°C 6-8 min. Fusion products were reamplified with Pfx50 (Invitrogen) to increase quantity, then purified using the Qiaquick PCR Purification Kit (Qiagen). Recipient strains grown 1 day on CBA were transformed with 500 ng of the final amplicon using natural transformation [42,43] followed by selection for 7-10 days on CBA containing 15 μg/ml chloramphenicol. To ensure allelic replacement, the resultant strains were evaluated by PCR of the genomic DNA using GoTaq (Promega) with primers specified in Table 1. PCR strategy and results are shown in Figure 1.

**Table 1:** Primer sequences.

*primers for allelic disruption*^a^		
CAT fwd [41]	GATATAGATTGAAAAGTGGAT	F5^b^

CAT rev [41]	TTATCAGTGCGACAAACTGGG	

cgtfwd	atggttattgttttagtcgtgga	

cgtM3	ATCCACTTTTCAATCTATATCatatggtggatatagcggtaatg	

cgtM5	CCCAGTTTGTCGCACTGATAAttaaaaacttgcaccctttatgt	

cgtrev	ctctgatcgcttcttcataaact	

pmifwd	atgaaaattaaaaatatcttactgagtggg	

pmiM3	ATCCACTTTTCAATCTATATCatctaaaccattagggctttcaatatac	

pmiM5	CCCAGTTTGTCGCACTGATAActttagtgaacgaggtagaaacaaac	

pmirev	ttttgtctgttaaaatcatcatcaat	

lpxE fwd	atgaaaaaattcttatttaaacaaaaattttgtgaaagc	

lpxEM3	ATCCACTTTTCAATCTATATCcccaaacgctgatcgttgat	

lpxEM5	CCCAGTTTGTCGCACTGATAAcgagcgcccttatggag	

lpxErev	ttaaggctttttggggcttgtaaa	

eptAfwd	ttggcatcattattccatctgaggt	

eptAM3	ATCCACTTTTCAATCTATATCgcaacaccccaaaaacaacgata	

eptAM5	CCCAGTTTGTCGCACTGATAAagcctgattaacgcctatgaca	

eptArev	ttactcttttttgtgtttaagcagatctaaagaa	

***additional primers for confirmation of gene disruption***		

G27_951fwd	agtgattcaagatggcgtgaaaa	F1

G27_953rev	ccaagctcaatcatttctttgtcttt	R1

G27_37fwd	cggcatggggatcaatcaag	F2

G27_39rev	ctcccgtcttgcccggtaac	R2

G2719fwd	gggcgataaaatcgtgtttca	F3

G2721rev	tcccctttatcgtttatgctaatga	R3

G2720fwd	cccaaactgagcgctaaca	F4

G2722rev	aagaaatttcaaggtataatagtttccaag	R4

^a^Respective gene numbers in public databases for 26695 and G27 are as follows: [*cgt: hp0421, G27_952*], [*pmi *(*rfbM*)*: hp0043, G27_38*], [*lpxE: hp0021, G27_20*], [*eptA: hp0022, G27_21*].

^b^Righthand column lists the brief primer designations used in Figure 1.

### Gastric colonization

Animal experiments were approved by the LSUHSCS Institutional Animal Care and Use Committee. Female Mongolian gerbils were maintained on ordinary diet *ad libitum*. To preserve motility, *H. pylori *strain SS1 was cultured overnight under microaerobic conditions in T75 flasks containing 40 mls of F12 medium with 0.4 mg/ml albumin and 0 or 50 μg/ml cholesterol. The motile planktonic bacteria were harvested by centrifugation and resuspended in isotonic saline. Colony forming units (CFU) were measured in these inocula by serial dilution and plating on CBA, and these measurements confirmed equal dosage of viable bacteria between the two growth conditions. Approximately 10^8 ^CFU per 30 μl were given orally to animals (n = 6 to 9 per group). Animals were euthanized 11 days later, and stomachs were removed and dissected. *H. pylori *present in gastric antrum homogenates were quantitated by serial dilution and plating on CBA containing 5-fluorocytosine (5 μg/ml), vancomycin (10 μg/ml), amphotericin B (5 μg/ml), bacitracin (30 μg/ml), polymyxin B (10 U/ml), and trimethoprim (10 μg/ml) [44]. Duplicate CFU determinations were made for multiple dilutions of each tissue sample.

### Whole-cell ELISA

Standard procedures [6,7,45], were adapted for the use of peroxidase conjugated secondary antibody. All antibodies were obtained from Calbiochem. Overnight cultures of bacteria were collected by centrifugation at 3500 × *g *for 10-15 min, washed in Dulbecco's phosphate buffered saline, and repelleted at 10,000 × *g *for 2 min, then resuspended in 15% glycerol/0.9% NaCl. The cell suspensions were assayed for protein content and stored at -20°C. Cell samples containing known amounts of protein were rapidly diluted into 50 mM sodium bicarbonate/carbonate pH 9.55 and dispensed immediately into wells of an ELISA plate (Costar #9017). Plates were sealed and refrigerated overnight, then blocked for 90 min in 3% bovine serum albumin dissolved in the wash buffer which consisted of 0.1 M sodium phosphate pH 7.4/0.1 M NaCl/0.1% w/v Tween-20. Primary antibody, monoclonal anti-Lewis X (Signet clone P12) or anti-Lewis Y (Signet clone F3), diluted 1:500 in wash buffer/1% BSA, was added for 2 hours, followed by four changes of wash buffer. The secondary antibody, a 1:2500 dilution of horseradish peroxidase-conjugated goat anti-mouse IgM in wash buffer/1% BSA, was added for 90 min, followed by four changes of wash buffer. The chromogenic substrate was 0.42 mM tetramethylbenzidine and 0.02% H_2_O_2 _in 50 mM acetate/citrate pH 5.5 [46]. After 15 minutes at room temperature, reaction was stopped with 1/5th vol 2.5 N H_2_SO_4_, and color change was measured in a plate reader at 450 nm. In negative controls omitting either primary or secondary antibody, or with *E. coli *strain HB101 substituted for *H. pylori*, color change was negligible (A<0.05). Levels of Lewis Y were negligible (A<0.1) in strain 26695 or 43504, as were Lewis X levels in SS1.

### Electrophoretic analyses of lipopolysaccharides

*H. pylori *cultures were collected as described above, and washed cell pellets were stored at -70°C. Cells were lysed in 60 mM Tris HCl pH 6.8 containing 2% SDS at 95-98°C for 10 min. Protein content was measured using the bicinchoninic acid assay (Pierce). Samples of cell lysates were adjusted to equal protein content (1 mg/ml), then proteolyzed in reactions containing (final) 60 mM Tris HCl pH 6.8, 0.67% SDS, and 0.67 mg/ml proteinase K at 60°C for 2 hours [47]. To eliminate electrophoretic artifacts due to the presence of lipid/detergent complexes, proteolyzed samples were extracted with hot phenol [48]. Control experiments verified that all LPS bands were recovered through the following extraction procedure qualitatively and without bias. Proteolyzed samples were mixed with 1 volume of 90% aqueous phenol and incubated at 70°C for 20 min. After cooling to 10°C for 1 min, the samples were centrifuged at 12,000 × *g *for 20 min at 10°C, and the aqueous phase collected. The phenolic phases were re-extracted with 1 volume of H_2_O at 70°C for 10 min, and the centrifugation repeated. The combined aqueous extracts were adjusted to 0.5 M NaCl and precipitated with 10 vol ethanol in the refrigerator overnight, then centrifuged at 20,000 × *g *for 20 min at 10°C and air dried. Purified LPS samples were redissolved in Laemmli sample buffer [49] at 95°C for 5 min. Samples were applied to 15% polyacrylamide/0.9% bis minigels containing 3.2 M urea with the Laemmli discontinuous buffer formulation [49], and a 5% stacking gel. After electrophoresis at 150 V for 75 min, gels were either fixed overnight for silver staining [50] or transferred to polyvinylidenedifluoride membrane using Tris/glycine transfer buffer [51]. Blots were blocked overnight in 3% bovine serum albumin and 0.03% NaN_3 _in the wash buffer described above for ELISA. Primary antibody (anti-Lewis X or anti-Lewis Y, 1:200) and secondary antibody (peroxidase-conjugated goat anti-mouse IgM, 1:1000) were diluted in wash buffer containing 0.5% BSA. Colorimetric detection used 3,3'-diaminobenzidine with cobalt enhancement [52]. Densitometry was performed with the public domain application Image J, available at http://rsb.info.nih.gov/ij.

## Results

**Figure 2:**
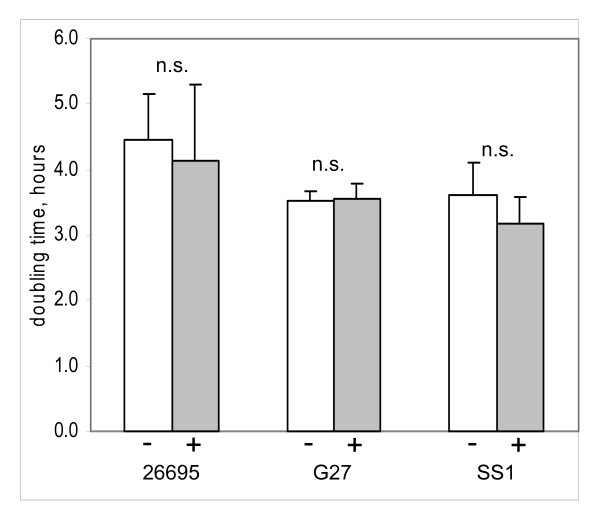
**Addition of cholesterol to the defined medium does not affect *H. pylori *growth rate**. Parallel cultures of each strain were grown overnight in F12/albumin (1 mg/ml) in the absence (open bars) or presence (shaded bars) of 50 μg/ml cholesterol. The initial population density was 2 × 10^6^/ml. Doubling times were calculated from the measured increase in biomass. Values shown represent the mean ± sd of five or more independent measurements. *n*. *s*. Student's two-tailed t-test for pairwise data showed no statistical significance.

**Figure 3:**
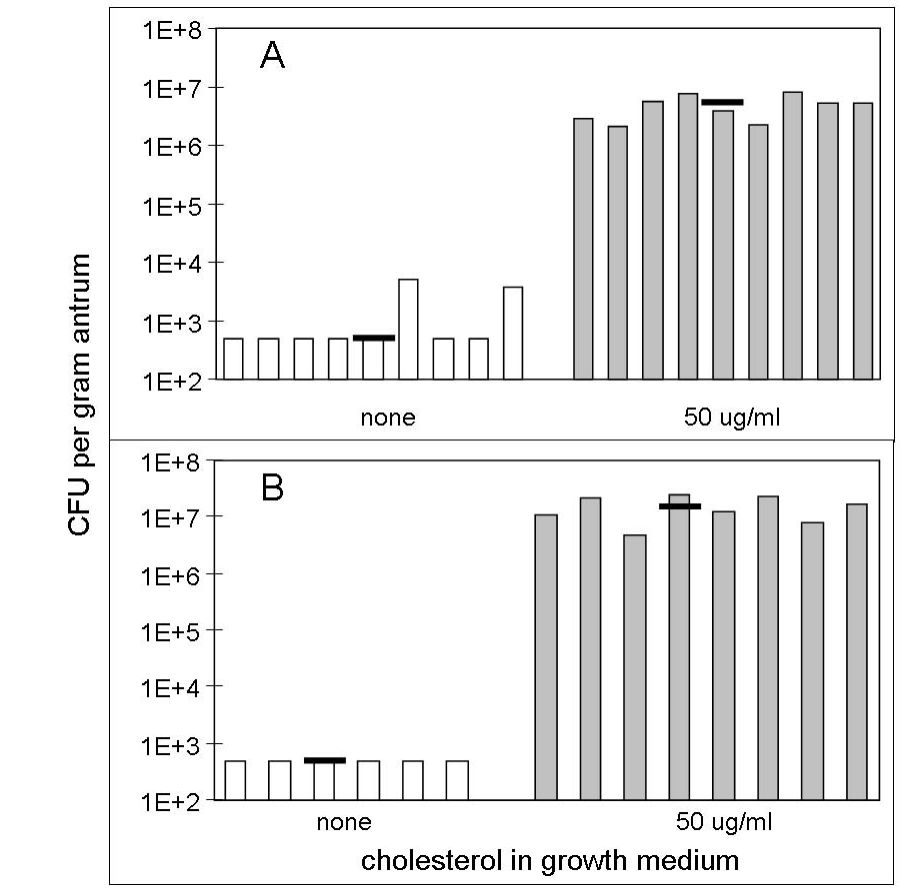
***H. pylori *grown without cholesterol fail to colonize gerbils**. *H. pylori *strain SS1 was grown overnight in defined medium containing 0 or 50 μg/ml cholesterol. Gerbils were orally inoculated with 3.5 × 10^8 ^CFU (experiment A) or 1 × 10^8 ^CFU (experiment B). *H. pylori *in gastric antrum were quantitated at 11 days. Each vertical bar represents the mean of duplicate determinations for one animal, and horizontal lines give the median for each treatment group. Where no colonies were recovered, values were recorded as 5 × 10^2 ^CFU/g tissue, the estimated limit of detection.

Little is known about the physiologic roles of cholesterol in *H. pylori*. To investigate responses of *H. pylori *to cholesterol, we adopted a defined, serum-free culture medium, F12 with 1 mg/ml albumin, in which this bacterium may be stably passaged [26]. This modest concentration of albumin boosts growth [25,26] and alleviates the tight adherence to culture surfaces that occurs in protein-free media [53]. In this defined medium, addition of 50 μg/ml cholesterol did not significantly alter the growth rate (Figure 2). The absence of growth effects under the chosen culture conditions was advantageous for investigation of the physiological importance of cholesterol in *H. pylori*. Thus, we were able to compare gastric colonization of gerbils by strain SS1 that had been cultured in the defined medium containing varied amounts of cholesterol (Figure 3). Eleven days after oral inoculation, *H. pylori *in gastric antrum were selectively plated and quantitated. Strikingly, gerbils were colonized only by the cultures grown in cholesterol-containing medium, but not by *H. pylori *grown in cholesterol-free medium (In each experiment, P < .0001 for comparison of log (CFU/g) between groups using Student two-tailed t-test). Therefore, cholesterol was an essential component of the growth medium in order to establish *H. pylori *infection in this animal model.

**Figure 4:**
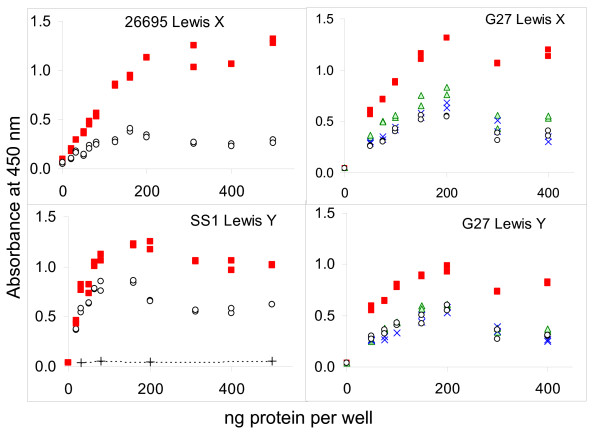
**Growth in cholesterol specifically enhances cell surface display of Lewis antigens**. Whole cell ELISA assays were performed on samples of *H. pylori *strain 26695 (upper left), SS1 (lower left), or G27 (upper and lower right). Parallel cultures were grown overnight in defined medium containing 130 μM of the following additions: circles, no addition; squares, cholesterol; triangles, β-sitosterol; X, taurocholate. Varying amounts of cell suspension corresponding to known amounts of cellular protein were applied to duplicate wells of ELISA plates, and immunoassayed for the presence of Lewis X or Lewis Y antigen as described in *Methods*. Negative control samples of *E. coli *HB101, or buffer-only blanks, fell on the dotted line. Absorbance readings for individual wells are plotted. Repeat experiments with three or more independently grown cultures have yielded essentially identical results.

**Figure 5:**
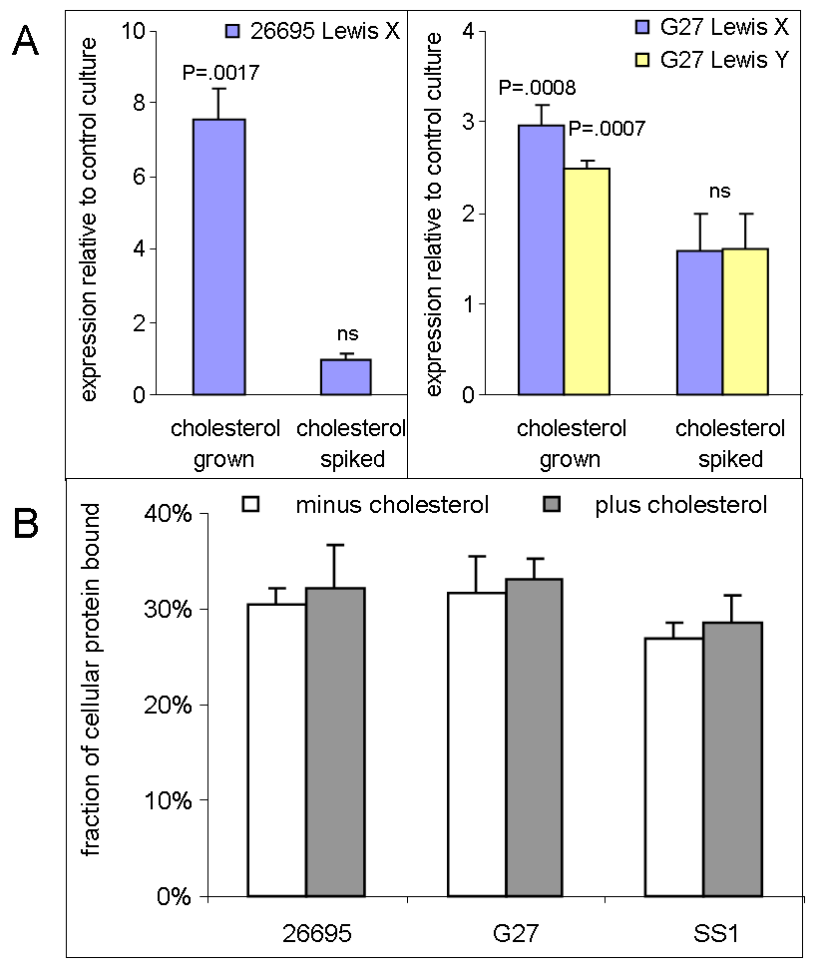
**ELISA control experiments**. **A. Spiking with cholesterol at the end of the growth period does not alter Lewis antigen expression**. Cultures of *H. pylori *were grown overnight in defined medium without (control) or with 50 μg/ml cholesterol (cholesterol grown). A third flask (cholesterol spiked) was grown in the absence of cholesterol, chilled on ice, and an equivalent amount of cholesterol was added before the cells were harvested. Lewis antigens were quantitated in duplicate by whole-cell ELISA, loading 300 ng cellular protein per well. Ratios for plus:minus cholesterol were calculated from average net absorbance readings in each assay, and the plot displays mean ratios ± sem for three to five independent ELISA runs. P values were calculated in two-tailed Student t-tests for the null hypothesis that the ratio equals 1. For comparisons labeled ns, P > .05. **B. Equivalent binding of cells to ELISA plates**. Samples of *H. pylori *that were grown in parallel cultures in the absence (white bars) or presence of 50 μg/ml cholesterol (grey bars) were applied to multiwell plates in the same manner as for Lewis antigen ELISA assays, adding 500 ng of cellular protein per well. Following overnight attachment, wells were washed twice with Dulbecco's phosphate-buffered saline, then protein in adherent cells was quantitated using the BCA reagent. Mean values ± sd of quadruplicate wells are shown.

Certain strains of *H. pylori *exhibited significant differences in adherence to culture vessels following passage in cholesterol, suggesting alterations in their cell surface properties (Hildebrandt & McGee, unpublished observations). For this reason, we decided to investigate lipopolysaccharides, which constitute the principal component of the cell envelope, and serve to present the biologically important Lewis antigens. We employed a well established whole-cell ELISA procedure to quantitate the predominant Lewis antigens, Lewis X and Y (Figure 4). In accordance with the literature [54,55], primarily Lewis X was detected in strain 26695, only Lewis Y was detected in SS1, and significant levels of both were detected in G27. In each case, absorbance readings were nonlinear with respect to sample load, an occurrence that is not unusual in ELISA assays [56], and that has been noted by other investigators using these same monoclonals [7]. Thus, in order to compare antigen levels in samples of *H. pylori *cultured in the absence or presence of cholesterol, we performed parallel titrations over a range of sample loadings varying from 20 to 500 ng of cell protein per well. These titrations reproducibly showed a marked increase in the amount of Lewis X and/or Lewis Y antigen detected on the cell surface when *H. pylori *strains 26695, SS1 or G27 were cultured in the presence of cholesterol (Figure 4). In replicate independent experiments, the mean cholesterol-dependent increases were statistically significant (Table 2). Comparable results have also been obtained for Lewis X in strain 43504 (data not shown). Spiking samples with cholesterol at the end of the growth period did not alter the amount of Lewis antigen detected by ELISA (Figure 5A). In another control experiment we verified for all four of these strains that the amount of cell protein bound to the wells was unaffected by growth in cholesterol (Figure 5B). The ELISA results thus established that increased surface expression of Lewis antigens was a legitimate biological response to cholesterol that occurred in all of the strains tested. This response was specific for cholesterol, because substitution of cholesterol in the growth medium with the structural analogs β-sitosterol or sodium taurocholate had no effect on Lewis X or Y expression by G27 (Figure 4, righthand panels, and Table 3). The Lewis antigen response to cholesterol still remained after disruption of the gene for cholesterol α-glucosyltransferase (*cgt*) in strain G27 (Table 2, and see below) and in 26695 (data not shown), ruling out the participation of α-glycoside metabolites of cholesterol.

**Table 2:** Enhancement of cell surface Lewis antigen expression by the growth of cultures in the presence of cholesterol.^a^

	*fold increase compared to parallel cholesterol-free culture*			
	
	Lewis X		Lewis Y	
	mean ± SEM (n)	*P value*	mean ± SEM (n)	*P value*
26695	4.32 ± 0.36 (6)	*0.0002*	not done	
SS1	not done		1.88 ± 0.08 (5)	*0.0004*
G27 wild type	2.85 ± 0.42 (8)	*0.0033*	2.22 ± 0.24 (8)	*0.0016*
G27 *cgt::cat*	3.69 ± 0.34 (5)	*0.0013*	2.88 ± 0.30 (5)	*0.0034*
G27 *lpxE::cat*	2.59 ± 0.50 (6)	*0.025*	2.47 ± 0.43 (7)	*0.014*

^a ^Lewis antigens were quantitated in replicate whole-cell ELISA analyses of paired samples grown in the presence or absence of 50 μg/ml cholesterol. The antigen load was 300 ng cellular protein per well. Ratios for plus:minus cholesterol were calculated from duplicate net absorbance readings in each assay, and ratios determined in five to eight independent ELISA runs were then averaged. P values were calculated in two-tailed Student t-tests for the null hypothesis that the ratio equals 1.

**Table 3:** Enhanced cell surface Lewis antigen expression is cholesterol-specific

	*fold increase compared to parallel cholesterol-free culture*			
	
	Lewis X		Lewis Y	
	mean ± SEM (n)	*P value*	mean ± SEM (n)	*P value*
cholesterol	2.96 ± 0.22 (5)	*.0008*	2.48 ± 0.10 (4)	*.0007*
β- sitosterol	1.80 ± 0.47 (4)	*0.19*	1.19 ± 0.13 (3)	*0.28*
taurocholate	0.64 ± 0.16 (4)	*0.12*	0.84 ± 0.20 (3)	*0.52*

Lewis antigens were quantitated in replicate whole-cell ELISA analyses of pairwise cultures of *H. pylori *G27 grown in the presence or absence of 130 μM cholesterol, or an equal concentration of β-sitosterol or sodium taurocholate. The antigen load was 300 ng cellular protein per well. Ratios for plus:minus cholesterol were calculated from duplicate net absorbance readings in each assay, and ratios determined in three to five independent ELISA runs were then averaged. P values were calculated in two-tailed Student t-tests for the null hypothesis that the ratio equals 1.

**Figure 6:**
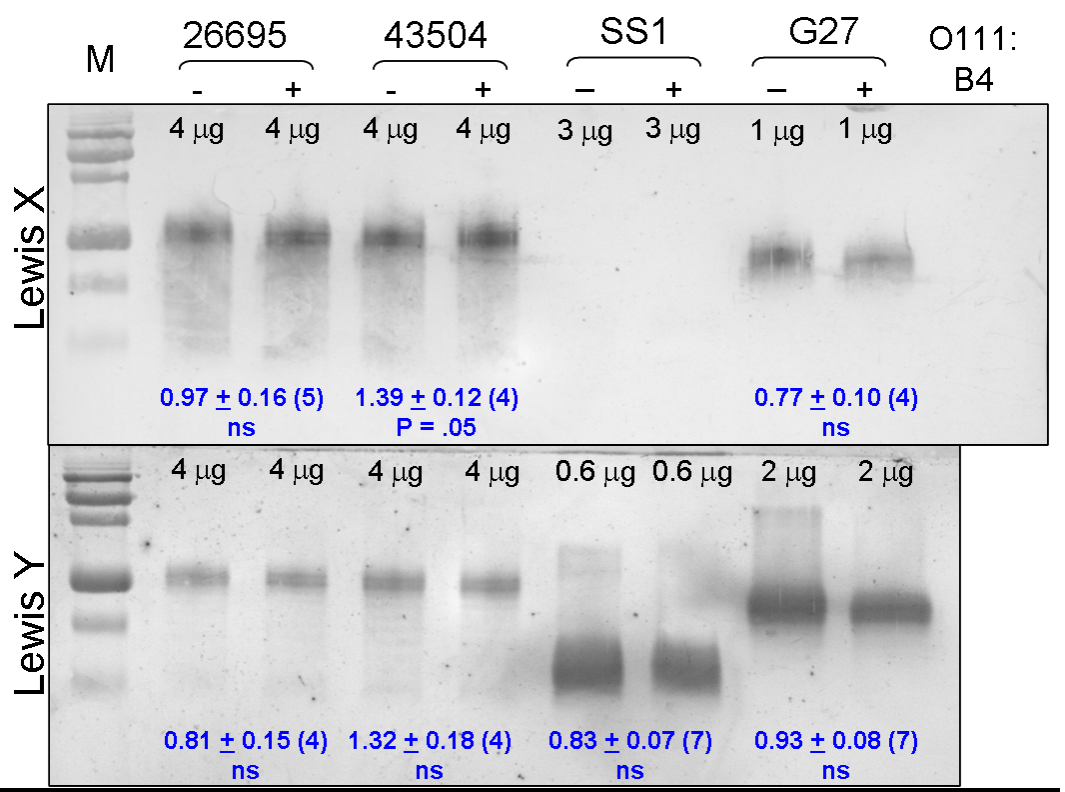
**Lewis X and Y antigen profiling by immunoblotting**. Samples of LPS isolated from parallel cultures grown in the absence (-) or presence (+) of 50 μg/ml cholesterol were resolved on 15% urea gels. Quantities loaded per lane, as μg of initial lysate protein, are given at the top of each lane. Following transfer, antigens were immunodetected with monoclonal antibodies specific for Lewis X (upper panel) or Lewis Y (lower panel). A representative example of each is shown. Side lanes contain prestained protein markers (M) or 400 ng of *E. coli *O111:B4 LPS. Antigenic signal appeared only in the O-chain regions of these *H. pylori *strains; blank areas have been cropped out accordingly. The immunoblots were independently replicated with several sample sets, and densitometry was used to quantitate antigen signal in each lane. Ratios for pairwise plus:minus cholesterol samples were calculated, and the mean ratios ± sem for (n) blots are given in blue. The null hypothesis that the ratio equals 1 was evaluated in a two-tailed Student t-test.

Detection of Lewis X and Y by immunoblotting with the same monoclonal antibodies produced a different result (Figure 6). In several attempts using this technique, we did not detect any cholesterol-dependent differences in Lewis X or Y levels, apart from a small increase in Lewis X in 43504 that was only marginally significant. The blotting procedure employed LPS samples extracted from cell lysates, and in principle should detect the entire cellular Lewis antigen pool, whereas the whole-cell ELISA method is designed to detect only that presented on the extracellular surface. The interesting difference in results between our ELISA analyses and immunoblots suggests a change in cellular compartmentation of the Lewis antigen depending upon the availability of cholesterol in the growth medium.

**Figure 7:**
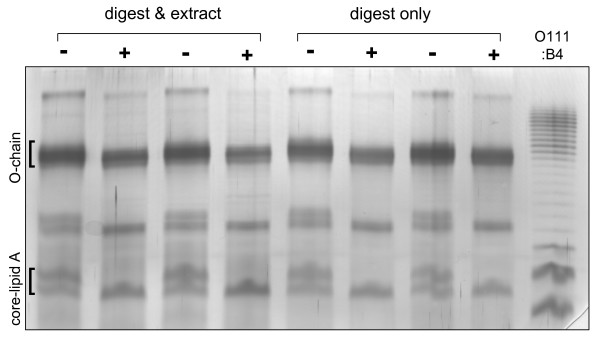
**G27 LPS species are quantitatively recovered in purified preparations, and respond to cholesterol in the growth medium**. In two independent experiments, parallel cultures of *H. pylori *strain G27 were grown overnight in defined medium without (-) or with (+) 50 μg/ml cholesterol. Cell lysates were digested with proteinase K, and portions of each lysate were further purified by hot phenol extraction and alcohol precipitation. Aliquots taken after digest only or after the extraction/precipitation procedure were resolved on a 15% urea gel. Each lane represents an amount of sample material derived from an equivalent amount of the initial cell lysate (2 μg protein). The reference lane contains 400 ng of LPS from *E. coli *O111:B4 as a silver staining control. No bands were selectively gained or lost in the workup following proteolytic digestion.

**Figure 8:**
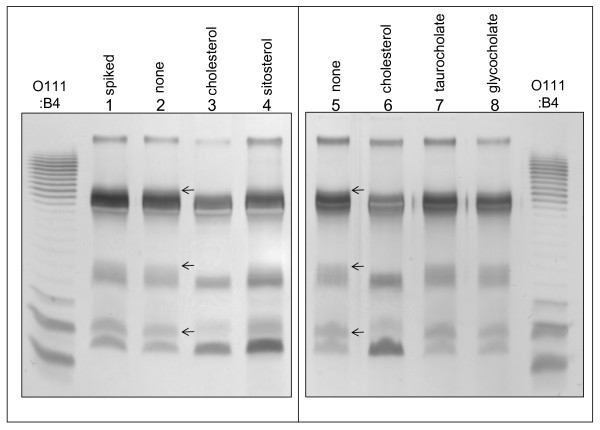
**LPS structure in *H. pylori *strain G27 responds specifically to growth in cholesterol**. In two independent experiments, parallel cultures of *H. pylori *strain G27 were grown overnight in defined medium. The growth media contained the following, each at 130 μM: lanes 1, 2, 5, no addition; lanes 3, 6, cholesterol; lane 4, synthetic β-sitosterol; lane 7, taurocholate; lane 8, glycocholate. At the end of the growth period the cultures were chilled on ice, and an equivalent amount of cholesterol was then added to sample 1. Cell lysates were adjusted to equal protein content, digested with proteinase K, and resolved on a 15% urea gel as described in *Methods*. Sample amounts loaded per lane correspond to 3 μg of cellular protein (lanes 1-4), or 2 μg (lanes 5-8). The indicated reference lane contains 400 ng of purified LPS from *E. coli *strain O111:B4. Arrows mark the specific bands that diminish in cholesterol-grown cultures.

In addition to Lewis antigen measurement, we directly compared the lipopolysaccharide profiles between parallel cultures grown in the presence or absence of cholesterol, using gel electrophoresis and silver staining. In all the *H. pylori *strains we have examined, LPS band profiles were identical between cultures grown in defined medium with cholesterol to that obtained in serum-containing medium or on blood agar (data not shown), and as expected [5,24,55,57] these profiles were highly strain-specific. On these gels, cholesterol-responsive LPS bands were most clearly resolved for the strain G27, a clinical isolate (Figures 7, 8). We confirmed that hot phenol extraction, which we included as an additional purification step, did not alter any of the bands seen on these gels (Figure 7). These analyses reproducibly showed that G27 cultures grown in cholesterol-free medium exhibited at least three additional LPS bands (Figure 8 lanes 2, 5, arrows) that were absent or strongly diminished when cholesterol was provided in the growth medium (lanes 3, 6). These bands included one in the core region, one in the O-chain region, and a band with intermediate migration on the gel. The responsive band in the core region (bottom arrow) was absent in plus-cholesterol samples, although on some gels a faint neighboring band could be seen which always migrated somewhat more slowly. Addition of cholesterol to the culture at the end of the growth period and prior to sample workup did not alter the LPS band profile (lane 1). Thus the observed band changes occurred biologically and not artifactually. This LPS response did not occur when the growth medium contained an equimolar amount of synthetic βsitosterol (lane 4), which differs from cholesterol by a single ethyl group in the alkyl side chain. Similarly, two bile salts which are well tolerated by *H. pylori*, taurocholate and glycocholate, did not affect LPS profiles (lanes 7, 8). Certain other cholesterol-like substances that we attempted to test proved toxic toward *H. pylori*; these included dehydroepiandrosterone, β-estradiol, and progesterone, as well as 5-β-coprostanol, a compound occurring in the human gut and differing from cholesterol by one double bond in the steroid nucleus. These findings together indicated that the observed LPS modification was strongly specific for cholesterol.

**Figure 9:**
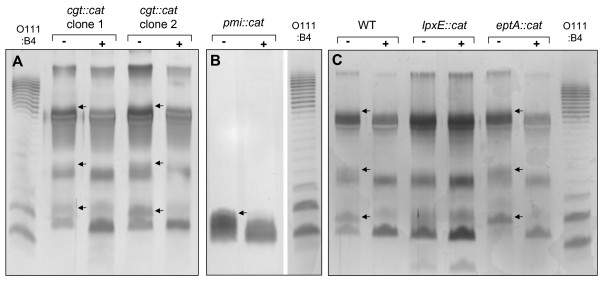
**Influence of selective gene disruptions on G27 LPS response to cholesterol availability**. In each experiment, parallel cultures of genetically altered G27 strains were grown overnight in defined medium without (-) or with (+) 50 μg/ml cholesterol. Cell lysates were adjusted to equal protein content, digested with proteinase K, and resolved on a 15% urea gel as described in *Methods*. Sample amounts loaded per lane correspond to 2 μg of cellular protein. Reference lanes contain 400 ng of purified LPS from *E. coli *strain O111:B4. **A**. LPS preparations from pairwise minus- and plus-cholesterol cultures of two individual *cgt::cat *G27 transformants. **B**. LPS from pairwise cultures of the O-chain-lacking *pmi::cat *G27 strain. **C**. LPS from pairwise cultures of wild type G27, or of isogenic *lpxE::cat *or *eptA::cat *strains.

The same LPS response to growth in cholesterol occurred in transformed G27 strains in which the cholesterol α-glucosyltransferase gene had been disrupted (Figure 9A). Therefore, α-glycoside metabolites of cholesterol were not required for the LPS changes observed on silver-stained gels.

We also investigated cholesterol responsiveness of LPS in a G27 *pmi::cat *strain lacking O-antigen chains (Figure 9B). As in wild type G27, this strain showed the presence of an additional, more slowly-migrating band in the core region that was diminished or lost upon growth in cholesterol. Likewise, *pmi::cat *strains of 26695 and SS1 also lacked O-chains, and also exhibited similar cholesterol-dependent band loss in the conserved LPS core region (data not shown). Since LPS species migrating in this region likely include only core oligosaccharide and lipid A moieties, we directed our attention to these components in trying to identify specific cholesterol-dependent structural modifications. We selectively disrupted two lipid A modification genes, either *lpxE *or *eptA*, encoding the lipid A 1-phosphatase and lipid A phosphoethanolaminetransferase, respectively [58]. Then, LPS profiles were compared in pairwise cultures of these mutated G27 strains grown in the presence or absence of cholesterol (Figure 9C). We found that the *eptA::cat *strain retained an LPS response to cholesterol that was even more distinct than in the wild type. In contrast, cholesterol-responsive bands were abolished in the *lpxE::cat *strain. These results implied that the aberrant bands which accumulated under conditions of cholesterol depletion in the wild type, but not in *lpxE::cat*, may represent forms of LPS in which the lipid A moiety has been dephosphorylated at the 1-position. It is also possible that, in these bands, the core may have undergone further modification subsequent to lipid A dephosphorylation (see *Discussion*).

**Figure 10:**
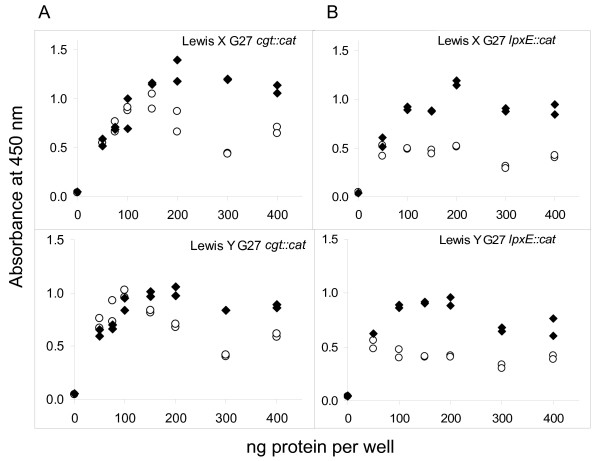
***H. pylori *G27 retain Lewis antigen response to cholesterol after disruption of *cgt *or *lpxE***. Whole cell ELISA assays were performed in duplicate on samples of *H. pylori *G27 *cgt::cat *(panel A) or *lpxE::cat *(panel B), which were cultured in parallel in the absence (open symbols) or presence of cholesterol (filled symbols). Absorbance readings for individual wells are plotted.

The LPS gel results described above (Figure 9C) contrasted with the outcome of whole cell ELISA analysis of the *lpxE::cat *strain. This mutant strain retained its capacity to respond to cholesterol availability with enhanced surface Lewis X and Lewis Y expression (Figure 10, Table 2), as did the *eptA::cat *strain (data not shown) and the *cgt::cat *strain (Fig. 10). These contrasting results show that the enhanced surface display of Lewis antigen in response to growth in cholesterol occurred independently of the structural modifications to the core/lipid A moiety seen on silver-stained gels.

## Discussion

In eukaryotic membranes, cholesterol modulates curvature and fluidity, and cholesterol-rich lipid subdomains influence numerous membrane functions, including signal transduction and transport activity [59], yet very little is known about the physiological roles of cholesterol among the prokaryotes that utilize it. In this study, we used chemically defined medium to begin to characterize these roles of cholesterol in *H. pylori*. Growth of *H. pylori *in the presence of cholesterol proved to be essential for gastric colonization in the gerbil, even though it is not necessary for growth *in vitro*. This colonization experiment was conducted under standard dietary conditions, where cholesterol should be abundant in gastric mucus [2,3,60]. Taking into account that *H. pylori *can also acquire cholesterol from the membrane of host gastric epithelial cells [35], our data would suggest that incorporation of cholesterol into the bacterial membrane prior to inoculation may facilitate early steps in gastric colonization that precede adherence to host epithelium, such as motility and/or acid resistance. Preliminary experiments have indicated that *H. pylori *grown in the presence of cholesterol are more resistant to acid and oxidative stresses than when cholesterol-depleted (DJM, unpublished observations). We propose that incorporation of cholesterol and/or cholesterol metabolites may strengthen the bacterial membrane against such stresses, protecting the bacterium from gastric acid prior to entry into the more pH-neutral gastric mucus layer. Once the epithelial layer has been colonized, host-derived cholesterol may then be utilized.

We have also presented evidence of a role for cholesterol in establishment of the normal lipopolysaccharide component of the cell envelope. Both Lewis antigen[12,14] and core oligosaccharide [13,61,62] contribute to *H. pylori *adherence and colonization. We have demonstrated here that cholesterol supports both increased display of Lewis X and Y antigens as well as the modification of LPS core/lipid A structure. These responses do not require cholesterol α-glycosides, but are nevertheless highly specific for cholesterol. No changes in Lewis antigen levels or in LPS profiles occurred when cholesterol was substituted by the structurally very similar β-sitosterol or other steroidal substances. There is experimental evidence for specific, protein-mediated cholesterol uptake by *H. pylori *[27], but no receptor has so far been identified.

In the clinical strain G27, specific LPS bands are observed under conditions of cholesterol depletion that do not occur upon growth in complex or defined media containing cholesterol. This suggests a requirement for cholesterol in the normal maturation of structure during LPS biosynthesis. Determination of the structure of LPS in G27, and identification of cholesterol-dependent changes to this structure, are currently in progress. We anticipate that cholesterol-dependent changes will likely be found within the core/lipid A portion of the LPS, because we also observed LPS band changes in isogenic strains that lack the O-chain. The loss of LPS O-chains by disruption of *pmi *was unexpected, as an NCTC11637 strain with a disruption in the same gene retained the O-chain [14]. We do not presently know why the LPS phenotype of the latter mutant differs from the *pmi::cat *strains that we generated using an allelic replacement strategy. Investigation of this matter is ongoing and will be the subject of another report. Directing our attention to the core/lipid A moieties, we attempted to identify LPS biosynthesis genes that, when disabled, would eliminate the observed LPS responses to cholesterol. We selected two genes, *lpxE *and *eptA*, that sequentially remove the lipid A 1-phosphate group and add 1-phosphoethanolamine [58]. Disruption of *eptA *did not affect cholesterol-dependent changes in the LPS profile, but disruption of *lpxE *eliminated this response to cholesterol. We propose that the LPS bands seen only under conditions of cholesterol depletion represent LPS with modified lipid A structure. This modified form could be 1-dephospholipid A, or a downstream form thereof (not including the 1-phosphoethanolamine form, which is ruled out by our *eptA::cat *results). While the entire sequence of LPS biogenesis has not been worked out in *H. pylori*, a ketodeoxyoctulosonic acid (Kdo) hydrolase activity has been detected in membrane fractions of *H. pylori *that removes the outermost of two Kdo residues subsequent to lipid A dephosphorylation [63]. Though to date no Kdo hydrolase gene has been identified, such a Kdo-modified derivative may be considered a candidate for the modified LPS. There may be other as yet unidentified downstream modifications as well. Positive assignment of the bands we observed is further complicated by the existence of a minor LPS form, in which lipid A bears an extra 4-phosphate group, and is hexa- rather than tetra-acylated [23]. Lipid A modifications are important because they strongly influence Toll-like receptor recognition, modulating innate immune responses [23,64].

In order to discuss potential mechanisms for these LPS effects, we must consider the architecture of LPS biosynthesis. In well-studied organisms such as *E. coli*, the numerous steps in LPS biogenesis take place in specific subcellular compartments, and require specific transporters to shuttle intermediates across the inner membrane, periplasmic space, and outer membrane [64,65]. Kdo_2_-lipid A is synthesized on the cytoplasmic face of the inner membrane, where the core oligosaccharide is separately assembled and then attached. This core-lipid A species must be flipped across the bilayer by the essential transporter MsbA. Modifications to lipid A are then carried out on the periplasmic face of the inner membrane. The O-chain is independently assembled in the cytoplasm on an undecaprenyl diphosphate carrier, transported across the inner membrane, and attached to the core-lipid A periplasmically. The multicomponent Lpt assembly transports full-length LPS across the outer membrane, where further trimming may occur. LPS biogenesis is species-specific, and for the case of *H. pylori *the picture is much less complete. Some but not all of the expected LPS transporter subunits have been identified in the genome [66,67]. Lipid A dephosphorylation and phosphoethanolamine addition have been assigned to the periplasmic compartment based on work in which these *H. pylori *genes were expressed in a temperature-sensitive MsbA mutant strain of *E. coli *[58]. Our data are consistent with periplasmic lipid A modification occurring independently of both O-chain addition and Lewis antigen addition, in keeping with the general model just described. This distinctly ordered process gives rise to a defined range of LPS molecules at the cell surface. Importantly, the LPS array can be remodeled in response to environmental conditions such as external pH [68,69].

How then might cholesterol modulate LPS biogenesis and modification? The lipid compositions of the inner and outer membranes of gram negative bacteria are specific and distinct [70], but little is known about the subcellular compartmentation of cholesterol in *H. pylori *or other prokaryotes. We propose that the presence of cholesterol is needed to establish the proper membrane composition and structure that permit the orderly building of nascent LPS as it transits across the inner membrane/periplasmic/outer membrane compartments. In this model, altered membrane composition may influence the activity of LPS biosynthetic enzymes embedded in the membrane, leading to improper LPS modification. Alternatively, cholesterol depletion may result in dysregulation of LPS transporter function due to alterations in membrane structure and composition. The dysregulated movement of LPS among inner membrane, periplasmic, and outer membrane compartments would then result in aberrant modifications to its structure. This scenario would be consistent with the observed discrepancy between whole cell Lewis antigen levels measured by immunoblot and cell surface levels measured by ELISA. That is, it is possible that under cholesterol-depletion the Lewis antigen-bearing LPS may be less effectively transported to the cell surface. Preliminary evidence indicates that membrane cholesterol may also influence certain ABC transporters and the ComB DNA transporter in *H. pylori *(Hildebrandt, Trainor and McGee, unpublished results). Thus, cholesterol may support a wider range of physiological processes in the bacterial membrane than is currently appreciated.

## Conclusions

We have demonstrated for the first time that cholesterol, though nonessential to growth of *H. pylori*, is nevertheless essential for gastric colonization in an animal model. We have further shown that cholesterol plays important roles in determining LPS structure as well as Lewis antigen expression, and that these biological effects are highly specific for cholesterol. LPS profiles of mutant strains lacking the O-chain retain responses to cholesterol availability, providing evidence for structural changes to the oligosaccharide core/lipid A moieties. Disruption of the lipid A 1-phosphatase gene, *lpxE*, eliminated the effect of cholesterol on LPS profiles, suggesting that aberrant forms of LPS that appear upon cholesterol depletion are dependent upon 1-dephosphorylation of lipid A. The roles of cholesterol in LPS structural modification and in Lewis antigen expression do not require α-glucosylation of cholesterol. Thus, cholesterol imparts these benefits independently of its previously reported role in resistance to host phagocytosis and T-cell responses, which require the alpha-glycoside metabolite of cholesterol [35]. Together these studies serve to emphasize the critical roles that cholesterol and its metabolites in the *H. pylori *membrane can play in host-pathogen interactions.

## Authors' contributions

DJM participated in animal experiments, oversaw development of the study, and edited the manuscript. EH contributed to study development, carried out molecular genetic and analytical work, participated in animal experiments, and drafted the manuscript. Both authors have read and approved the final manuscript.
